# What is the future of targeted therapy in rheumatology: biologics or small molecules?

**DOI:** 10.1186/1741-7015-12-43

**Published:** 2014-03-13

**Authors:** Attila Mócsai, László Kovács, Péter Gergely

**Affiliations:** 1 Department of Physiology, Semmelweis University School of Medicine, 1094 Budapest, Hungary; 2 MTA-SE “Lendület” Inflammation Physiology Research Group of the Hungarian Academy of Sciences and the Semmelweis University, 1094 Budapest, Hungary; 3 Department of Rheumatology, University of Szeged Faculty of Medicine, 6725 Szeged, Hungary; 4 Translational Medicine Autoimmunity, Novartis Institutes for Biomedical Research, CH-4002 Basel, Switzerland

**Keywords:** Rheumatoid arthritis, Biologic therapies, TNF antagonists, Small molecule therapeutics, Kinase inhibitors, Tofacitinib

## Abstract

**Background:**

Until late in the 20th century, the therapy of rheumatic diseases relied on the use of drugs that had been developed through empirical approaches without detailed understanding of the molecular mechanisms involved. That approach changed with the introduction of biologic therapeutics at the end of the 20th century and by the recent development of small-molecule inhibitors of intracellular signal transduction pathways. Here we compare and discuss the advantages and disadvantages of those two groups of targeted anti-inflammatory therapeutics.

**Discussion:**

TNF-blocking biologic agents were introduced into the therapy of rheumatoid arthritis and other autoimmune and inflammatory diseases in the late 1990s. Further biologic agents targeting cytokine networks or specific lymphocyte subsets have since been added to the armamentarium of anti-rheumatic therapy. During the last few years, another wave of novel discoveries led to the development of a new class of small molecule anti-inflammatory compounds targeting intracellular signal transduction molecules, such as tyrosine kinases. In all those cases, the specific targets of the drugs are well defined and significant knowledge about their role in the disease pathomechanism is available, qualifying them for being targeted therapeutics for inflammatory rheumatic diseases. While both groups of targeted therapeutics offer significant clinical benefit, they clearly differ in several aspects, such as the localization of their targets, their route of administration and target specificity, as well as technical details such as manufacturing procedures and cost basis. In this debate paper, we compare the advantages and disadvantages of the two different approaches, aiming to shed light on the possible future of targeted therapies.

**Summary:**

Biologic therapeutics and small-molecule inhibitors both have significant advantages and disadvantages in the therapy of rheumatic diseases. The future of targeted therapies is one of the most exciting questions of current rheumatology research and therapy.

## Background

Inflammatory rheumatic diseases such as rheumatoid arthritis (RA) or systemic lupus erythematosus (SLE) are severe and chronic diseases that affect a significant proportion of the population, cause deterioration of the quality of life and place a major burden on health care systems worldwide. The last several decades have witnessed significant improvement in the therapy of these diseases. However, there is an ongoing need for the development of newer, more efficient and more cost-effective therapeutic applications. The aim of this paper is to compare the benefits and drawbacks of two of the recent directions of targeted therapies in rheumatology: biologic therapeutics and small molecule inhibitors.

**Figure 1:**
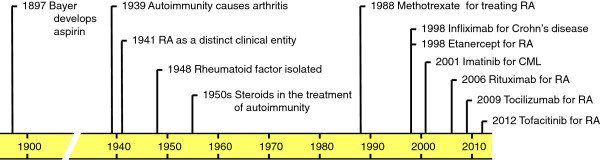
**Timeline of rheumatoid arthritis research and therapy.** RA, rheumatoid arthritis; CML, chronic myelogenous leukemia.

Steroids and non-steroidal anti-inflammatory drugs were introduced into the therapy of inflammatory rheumatic diseases around the middle of the 20th century (Figure 1) and provided symptomatic improvement and pain relief. However, they had significant side effects and, maybe more importantly, they failed to prevent disease progression and the development of a debilitating disease course. The identification of a beneficial effect of aminopterin antimetabolites led to the introduction of the chemotherapeutic agent methotrexate to the therapy of inflammatory rheumatic diseases in the late 1980s (Figure 1). The major advantage of methotrexate and related compounds (collectively termed disease-modifying anti-rheumatic drugs or DMARDs) was that they significantly delayed the progression of the diseases. Although methotrexate still remains the first line therapy for inflammatory rheumatic diseases, its cytotoxic (for example, hepatotoxic) nature and/or partial efficacy limit its use.

All of the above therapeutics were developed using an empirical approach without detailed understanding of their mechanism of action and therapeutic targets. However, the last decades have witnessed an explosion of our understanding of the inflammatory process and the molecular pathways involved. This led to a new approach of drug development whereby targeted therapies are developed by directly targeting molecules thought to be involved in the inflammatory process. A major approach for the development of targeted therapeutics has been the application of monoclonal antibody technologies for therapeutic purposes.

The first targeted therapeutics in immune mediated diseases were antibodies and related molecules interfering with the function of tumor necrosis factor-α (TNF-α or TNF), a major cytokine involved in various aspects of the autoimmune and inflammatory process [1]. The first TNF-antagonists against inflammatory diseases were infliximab (first approved for the therapy of Crohn’s disease) and etanercept (first approved for rheumatoid arthritis), both of them receiving marketing authorization in 1998 (Figure 1). Infliximab, as well as adalimumab, a third agent targeting the TNF-α cytokine, were later also approved for rheumatoid arthritis. Given the biologic, rather than chemical origin of these molecules, they have been termed biologic therapeutics. The major advance made by biologic agents was recognized by the 2003 Lasker award in clinical medicine given to Marc Feldmann and Ravinder Maini for ‘the discovery of anti-TNF therapy as an effective treatment for rheumatoid arthritis and other autoimmune diseases’ All of the above three drugs are proteinaceous molecules with antibody-related features. However, while infliximab and adalimumab are true monoclonal antibodies (infliximab is an engineered humanized mouse monoclonal antibody whereas adalimumab is a fully human antibody), etanercept is a fusion protein of the Fc region of a human immunoglobulin G (IgG) antibody linked to the extracellular portion of the human p75 TNF-receptor.

The initial advance with anti-TNF biologic therapeutics opened up new avenues for targeting other proinflammatory targets by biologic agents (Figure 1). Currently approved biologic agents for the treatment of rheumatoid arthritis include rituximab, a chimeric antibody targeting the CD20 receptor and, thus, leading to B-cell depletion; tocilizumab, a humanized mouse monoclonal antibody against IL-6; abatacept, a T-cell-blocking Fc-fusion protein of the extracellular domain of CTLA-4; and anakinra, an IL-1 receptor antagonist blocking the IL-1 receptor pathway [2]. There are also a large number of other biologic agents currently in advanced clinical trials for the therapy of rheumatoid arthritis [2,3] and other immune mediated diseases.

The fact that all of the above biologic agents are of a proteinaceous nature (mainly antibodies or antibody-related molecules) determines several important features, such as route of administration, half-life, localization of the target, manufacturing standards, and so on (Table 1). Therefore, despite their rather different therapeutic targets, these biologic agents are considered a unique group of therapeutics in rheumatology.

**Table 1:** Key characteristics of biologics versus small molecules

	**Biologics**	**Small molecules**
**Chemical composition**	Protein	Organic small molecule
**Structure**	Known sequence, variable three-dimensional structure and glycosylation	Well-defined structure
**Molecular weight**	>1 kDa	<700 Da
**Stability**	Protease and heat-sensitive	Mostly stable
**Administration**	Parenteral	Oral
**In vivo half-life**	(Usually) Long	(Usually) Short
**Target**	Extracellular	Intracellular
**Mechanism of action**	(Usually) Blocking, depletion	(Usually) Enzyme inhibition
**Specificity**	High	Low/variable
**Manufacturing cost**	High	Low/variable
**Degradation**	Catabolism	Metabolism
**Generics**	Biosimilar	Identical

The table highlights selected typical key characterisitics of biologic therapeutics and small-molecule anti-rheumatic agents. Specific drugs may deviate from the typical features. Further differences, such as costs and standards of manufacturing, regulatory requirements, registration path, supply chain logistics, and so on have been omitted.

During the last few years, an entirely different direction for developing novel anti-inflammatory agents has emerged. This was mainly driven by the tremendous success of targeting intracellular tyrosine kinases in cancer therapy. One of the prime examples of a breakthrough in oncology was the development of imatinib, an inhibitor of the Abl tyrosine kinase, for the targeted therapy of chronic myelogenous leukemia (Figure 1), a disease caused by the emergence of the BCR-Abl fusion protein through a unique chromosomal translocation [4]. Additional kinase inhibitors have also proved to provide significant clinical benefit in solid tumors [5]. Those developments also generated a vast experience with therapeutic targeting of intracellular kinases. However, kinases are not only involved in malignant processes but they also play critical roles in non-malignant (for example, inflammatory) diseases [6]. Importantly, tyrosine kinases, such as Jak-family kinases [7], the Syk tyrosine kinase [8] or Src-family kinases [9], are also critically involved in immunological processes leading to the development of autoimmune diseases. This has led to the development of small-molecule inhibitors against various protein and lipid kinases for the treatment of autoimmune and inflammatory diseases.

One of the first kinase inhibitors to be developed for rheumatoid arthritis was tofacitinib, a highly selective inhibitor for Jak-family kinases, intracellular molecules involved in signal transduction by various cytokine receptors [7]. Tofacitinib was approved by the Food and Drug Administration (FDA) for the treatment of rheumatoid arthritis in late 2012 (Figure 1), as the first oral anti-rheumatic therapy since the era of biologic agents [10]. Fostamatinib, an inhibitor of the Syk tyrosine kinase which is involved in signaling by various receptors on hematopoietic lineage cells [8], also showed promising therapeutic effects in Phase IIb clinical trials [11] although its further clinical development is in question due to disappointing Phase III trials. Despite initial enthusiasm, targeting the p38 MAP kinase pathway did not prove to be a viable therapeutic option in rheumatoid arthritis [12]. In addition, a number of other small molecule anti-rheumatic agents are in advanced clinical trials for the treatment of rheumatoid arthritis [13]. Although kinase inhibitors are among the most widely explored anti-inflammatory drug candidates, small molecules acting on non-kinase targets such as calcineurin, mTOR, adenosine receptors or ion channels may also provide significant therapeutic benefit.

While a vast knowledge on biologic agents has been obtained during the last 15 years, we are just beginning to collect significant clinical experience with small-molecule kinase inhibitors. It is already clear that small-molecule inhibitors have their own unique features related to targets, route of administration, specificity or manufacturing, all of which are quite different from biologic therapeutics (Table 1). Nevertheless, both groups have major benefits and the outcome of their long-term comparison will be one of the most exciting questions of clinical rheumatology in the next several years.

## Discussion

### Beneficial aspects of biologic therapies

Biologic therapies have successfully been introduced into the treatment of several inflammatory rheumatic diseases, including rheumatoid arthritis (RA), ankylosing spondylitis (AS), psoriatic arthritis (PsA), SLE, juvenile idiopathic arthritis (JIA), osteoporosis and ANCA-associated vasculitis. Currently, monoclonal antibodies or fusion proteins targeting TNF-α, interleukin-6 receptor (IL-6R), B-lymphocytes (the CD20 cell surface marker), interleukin-1 (IL-1), B-lymphocyte stimulator (BLyS), and CD28-CD80/CD86 co-stimulation have been registered for the treatment of patients with inflammatory rheumatic diseases refractory to conventional immunosuppressive treatment, and the list of promising targets and new indications seems to be growing rapidly [2,3].

#### Established long-term efficacy

A key argument in favor of biologics is their outstanding efficacy that has revolutionized the treatment of the above-mentioned diseases, leading to novel treatment paradigms. Every novel anti-rheumatic drug is benchmarked against the high standards set by biologics.

Anti-TNF therapy significantly improves the disease activity of RA, AS or PsA in the majority of the patients compared with conventional DMARDs [14,15], although the benefits become evident only when the biologic is combined with methotrexate, apart from the IL-6R blocker tocilizumab, which appears to be superior to methotrexate even in monotherapy [16]. As many as 40% of the anti-TNF-treated patients can reach a complete remission of the disease, and the benefits of the drugs persist at least as long as five years in the majority of the patients [15]. Long-term remission and even drug-free remission has become a reality in selected patients, especially if the active treatment is started early in the disease course [17]. Consequently, treatment guidelines and diagnostic criteria have recently been updated in order to facilitate early diagnosis and tight control of disease activity [18]. In addition, biologics have been consistently found to reduce, or often halt, the radiographic progression of RA, to preserve the functional status of the involved joints, to reduce fatigue, to control extra-articular manifestations (including accelerated atherosclerosis, the major cause of death in RA patients) and to enable the patients to preserve their ability to work. As all these benefits have been proven to persist during several years of follow-up, the cost-effectiveness of these otherwise expensive drugs has been calculated as acceptable.

#### Biologics are highly selective for their target molecules

The therapeutic effect of biologics relies on highly specific protein-protein interactions and, therefore, they are highly selective for their targets. It has been emphasized that specific targeting of a single molecule that has a key pathogenic role in a particular disease increases the efficacy of the drug and reduces the risk of side effects. Oral Jak inhibitors, on the other hand, have multiple targets, as listed in Table 2. Tofacitinib has been shown to block Jak1, Jak3 and, to a lesser extent, Jak2 [19]. While a balanced blockade of several cytokines listed in Table 2 may prove to be beneficial, the activity of a number of other biologically active agents is also modulated by tofacitinib (Table 2). These include haematopoietic factors, growth hormone and growth factors, and substances interfering with lipid metabolism, energy homeostasis, vasoregulation and haemostasis. Some of these interferences (for example, anemia) may have clinical consequences as indicated by data from randomized clinical trials using tofacitinib in which neutropenia and anemia (usually mild) occurred more often in the tofacitinib-treated patients than in the placebo group [20]. These effects, likely related to the broad targeting of Jaks, require clinical monitoring. In addition to adverse effects related to other biological functions of the primary targets (for example, Jak kinases), off-target effects may also represent a major problem for small-molecule inhibitors. There are, however, strategies that may predict off-target effects in an early phase of drug development, such as genome-wide transcriptome analysis, other *in vitro* and *in vivo* assays or new design of future clinical trials [21-23].

**Table 2:** The use of Jak-family kinases by cytokines and other intercellular mediators

**Ligand**	**Jak-kinase**
IL-6, IL-11, CNTF, CT-1, LIF, OSM, IL-27 (EBI3 + p28), IL-31, IL-35 (p35 + EBI3)	Jak1, Jak2, Tyk2
**G-CSF**, IL-12 (p40 + p35), **angiotensin**	Jak2, Tyk2
**Leptin, GM-CSF**, IL-5, IL-3, IL-23 (p40 + p19), **serotonin, α-thrombin**	Jak2
Chemokines	Jak2, Jak3
IL-2	Jak1, Jak2, Jak3
IL-4, IL-9, IL-7, IL-15, IL-21	Jak1, Jak3
IL-13	Jak1, Jak2, Tyk2
IL-19, IL-20	Jak1, ?
IL-22, IL-26, IL-28A, IL-28B, IL-29, interferon (IFNα/β), IL-10	Jak1, Tyk2
IL-24	Jak1, ?
**GH, Epo**	Jak2
**Thrombopoetin**	Jak2, Tyk2
IFN-γ, **PDGF**	Jak1, Jak2
**TLSP**	Jak1, possibly Jak2
**EGF**	Jak1

Substances that may be involved in off-target effects of Jak-family inhibitors are highlighted in bold. CNTF, ciliary neurotrophic factor; CT-1, cardiotrophin-1; EGF, epidermal growth factor; Epo, erythropoietin; G-CSF, granulocyte colony stimulating factor; GH, growth hormone; GM-CSF, granulocyte-macrophage colony stimulating factor; IL, interleukin; LIF, leukemia inhibitory factor; OSM, oncostatin-M; PDGF, platelet-derived growth factor; TLSP, thymic stromal lymphopoietin.

Of note, anemia was also more common in the adalimumab-treated subjects [24]. Clinically significant neutropenia and associated infection are rare with anti-TNF therapies and also with rituximab, but the regular control of blood count is advisable. In contrast, neutropenia occurs relatively frequently during the IL-6R blocker therapy (with a frequency of 29% and 33% in two randomized controlled trials (RCTs) [16,25], but high-grade neutropenia or significant infectious events are rare. Hemoglobin levels typically normalize quickly after the initiation of anti-TNF therapy, and even faster during tocilizumab treatment.

#### Beneficial cardiovascular effects of biologics

Anti-TNF agents have proven to reduce all-cause cardiovascular morbidity and mortality [26]. This effect is likely linked to changes in lipid metabolism; however, its mechanism is currently not fully understood. Total cholesterol, as well as both low-density lipoprotein (LDL) and high-density lipoprotein (HDL) levels typically decrease during an active inflammatory process in rheumatoid arthritis, but rise again once the acute phase response is suppressed by an effective therapy [27]. In this context, lipid levels display an inverse correlation with C-reactive protein (CRP) levels, a widely used marker of acute phase response. In fact, a persistently elevated CRP level has been found to closely correlate with cardiovascular risk, and the normalization of CRP in response to therapy is an indicator of lower atherogenic risk. Some investigators have found levels of LDL to rise and HDL to decrease during infliximab therapy [28], a phenomenon called the ‘lipid paradox’ (indicating reduced cardiovascular risk despite an increased LDL to HDL ratio) [27], whereas others have demonstrated that HDL levels and the atherogenic index are unchanged. More detailed analyses have revealed that the Apo B/A-I ratio improves and macrophage inhibitory factor levels decrease during adalimumab treatment, and that the capacity of HDL to block the oxidation of LDL and paraoxonase-1 activity increases during anti-TNF therapy [29]. All of these findings support the beneficial effects of anti-TNF treatment on lipid metabolism and atherosclerotic risk.

TNF-α is also known to cause endothelial activation, including the up-regulation of adhesion molecules, and it increases the production of coagulation factors and enhances platelet activation [30]. Endothelial dysfunction is seen as a major pathogenic factor in RA-associated cardiovascular morbidity with an important role for TNF-α [31,32]. TNF-α also contributes to insulin resistance, a phenomenon that occurs more often in RA patients than in matched healthy subjects or osteoarthritis patients [33].

Both tocilizumab, a biologic agent targeting IL-6, and the small molecule tofacitinib have been shown to significantly affect total cholesterol, LDL and HDL levels, although the overall effect on cardiovascular risk has yet to be determined [24,34,35]. Findings suggest that tocilizumab could improve the arterial stiffness with comparable efficacy to anti-TNF agents; however, the net effect of tocilizumab and tofacitinib on long-term cardiovascular morbidity has yet to be elucidated.

#### Suitability for renal or hepatic impaired patients

Biologic therapies can be administered in patients with seriously impaired renal function. No differences have been found in the pharmacokinetics of etanercept between patients on hemodialysis and those with normal renal function. Obviously, these agents are not removed by hemodialysis or peritoneal dialysis, and, consequently, they have been found to be safe and effective in these patients [36]. In contrast, there are no such data available for novel small molecules, such as tofacitinib, in patients with severe renal failure. The dose of tofacitinib must be reduced in patients with moderate renal insufficiency according to the current FDA label. A proportion of patients with impaired renal function may not be appropriate candidates for Jak-inhibitor therapy.

Liver enzyme elevations (>3× upper limit of normal) were more common with tofacitinib than with adalimumab therapy [20]. Abnormal liver function is not a contraindication to anti-TNF or rituximab treatment, and hepatic enzyme elevation is rare, usually mild and transient during these therapies. In contrast, the dose of tocilizumab should be reduced in patients with moderate liver enzyme elevation, and the drug is not recommended for patients with severe hepatic impairment according to the FDA label. It should be noted that similar restrictions apply to the use of the IL-6R blocker tocilizumab, as this agent also causes liver enzyme elevations in a significant proportion of patients.

#### Intravenous infusion ensures maximum compliance

It has been stressed that one potential advantage of small molecules is their oral administration route; however, arguments may also favor parenteral administration. Studies comparing intravenous and oral bisphosphonate anti-osteoporotic regimens have suggested a poorer compliance with once weekly oral administration as compared with a tri-monthly or yearly intravenous route, which may partly be responsible for the lower efficacy of the oral agents [37]. A better compliance may be expected with injected therapies than with twice daily tablets in a certain proportion of patients. Apart from compliance, parenteral administration of methotrexate has been shown not only to cause fewer gastrointestinal adverse effects but also to have higher efficacy and a faster onset of action [38].

#### Biologics may be well suited for individualized therapy

As RA and most of the autoimmune rheumatic diseases are also of a heterogeneous nature in terms of therapeutic response, the identification of biomarkers with a potential to predict the response to the particular agent is of high priority. Based on theoretical considerations, differential responses to drugs targeting one single molecule may better identify disease subsets than a molecule with pleiotropic activity. Several parameters have been identified with a potential to predict response to anti-TNF or B-cell-depleting therapies [39], and some of the identified biomarkers (such as genetic polymorphisms or levels of autoantibodies) may prove to be useful in the identification of patient populations with optimal response to biological therapies. However, it should be noted that truly predictive biomarkers have so far mainly been identified for small-molecule inhibitors in oncology; therefore, small molecule inhibitors may be equally well suited for the individualized therapy of inflammatory diseases.

### Beneficial aspects of small-molecule therapeutics

The introduction of biologics and substantial additional efforts aimed at understanding autoimmunity and inflammation at the molecular level has not only revolutionized the therapy of rheumatic diseases but facilitated the identification of key pathogenic molecules in autoimmune diseases. A better understanding of the molecular nature of immune cell receptors as well as the intracellular signaling pathways downstream of these receptors has led to novel approaches to target various players in disease pathogenesis. Until recently, however, the success of small molecule targeted therapeutic approaches in rheumatology has not achieved the level of biologics, mainly due to off-target toxicity (non-selectivity), inefficacy, or unfavorable risk/benefit ratios. This is in contrast to the experience in cancer therapy where small molecule-based targeted therapies (such as BCR-Abl inhibitors) have been used successfully for the treatment of chronic myeloid leukemia and other hematological malignancies.

How can small molecules contribute to the therapeutic armamentarium of rheumatic diseases that is currently dominated by biologics? For this, one should take into account the key differences between small molecules and biologics (Table 1). In particular, small molecules: 1) are typically administered orally; 2) have a higher likelihood for off-target effects (based on non-selectivity); and 3) have the ability to directly target intracellular signaling pathways. This section reviews these three key points to underscore why novel small molecules may also have a promising future in rheumatology.

#### Oral administration is a major advantage for small molecules

Despite the outstanding efficacy and good safety/tolerability profile of the currently used biologics in rheumatology, the requirement for long-term injections or infusions can impose a burden on patients leading to reduced adherence. Needle phobia is not uncommon: twenty-two percent of participants in the general population reported a fear of needles in a recent survey [40]. In multiple sclerosis, for which no effective oral treatment was available until recently, injection anxiety or needle phobia could prevent patients from self-injecting their biologics [41]. Misunderstanding about the risks of self-injection or a lack of knowledge about how best to manage injection pain and side effects may also result in an inability to self-inject [41].

Advances in injection technology, such as the introduction of auto-injectors have improved patient satisfaction and reduced the incidence of injection-site reactions; however, patients’ resistance to self-injection still exists [41]. According to the RAISE (Rheumatoid Arthritis: Insights, Strategies and Expectations, 2009) survey, about 25% of survey participants with RA currently treated with a subcutaneous injection rely on caregivers or healthcare providers to administer the medication, and 24% of patients who self-inject experience pain upon injection and 20% experience irritation at the injection site [42]. Injection-site reactions or patients’ negative feelings towards any therapy administered via needles may negatively affect patient acceptance of treatment and often result in non-compliance.

Non-adherence to a treatment regimen is a prevalent and major problem of patients with chronic disorders. Approximately half of the patients with a chronic disease have problems following their prescribed regimen to the extent that they are unable to obtain the desired clinical benefit, and poor adherence attenuates optimal clinical benefit [43]. The World Health Organization has recognized the lack of adherence as a major problem in the management of chronic diseases and concluded that improving adherence would have an even more beneficial effect on health outcome than improving the efficacy of specific treatments [44]. It is often assumed that the population is generally adherent but in many chronic conditions, on average, only about half of the patients comply with care recommendations over the long term [45].

Overall, oral therapies with new modes of action have the potential to expand the current treatment repertoire, increase patient satisfaction and adherence, and thereby improve efficacy. Patients express greater satisfaction with the convenience of oral therapies, and oral medications are known to be preferred to injected therapies that have similar efficacy. Thus, treatment efficacy can be improved simply through its effect on adherence.

#### Broader target selectivity can be of therapeutic benefit for small molecules

The experience in cancer therapy established the principle that kinase inhibitors do not need to be absolutely specific to be clinically useful; off-target effects may even be therapeutically beneficial. The competitive inhibition of the BCR-Abl tyrosine kinase leads to inhibition of proliferation, restoration of cell cycle control, induction of apoptosis and reversal of genetic instability in BCR-Abl dependent cells *in vitro*[46]. Based on this mode of action, BCR-Abl inhibitors such as imatinib or next generation sunitinib, dasatinib and nilotinib can induce remission in more than 90% of patients in the early stages of chronic myelogenous leukemia (CML). Imatinib, however, is not selective for BCR-Abl and also inhibits the PDGFR kinase and KIT receptor tyrosine kinase, thereby expanding its therapeutic utility to other diseases, for example, gastrointestinal stromal tumors [47] or idiopathic hypereosinophilic syndrome [48]. In addition, the effect of dasatinib is likely, at least in part, mediated by its inhibitory effect on Src-family kinases. Further potential benefit of broad(er) targeting may be the simultaneous modulation of several cytokine pathways in the same disease (for example, by Jak inhibition in RA) exerting a stronger therapeutic effect (Table 2).

Small molecules are often associated with adverse reactions due to off-target effects. However, biologics with very high specificity can also cause severe adverse events. For example, an increased risk of serious tuberculosis and other opportunistic infections has been reported with TNF-blocking agents across various studies, and that effect is likely directly related to the target molecule [49]. In addition, the effect of certain biologics (such as the B-cell-depleting rituximab) is mediated by depleting the cellular lineage expressing the target molecule; in that case, a wider functional consequence of biological therapy is expected. Small-molecule Jak inhibitors have been shown to adversely impact serum lipid profiles; however, pro-atherogenic lipid changes and decreased hepatic LDL receptor expression were also demonstrated with tocilizumab in RA [34]. The net effect of these drugs on morbidity and mortality has to be confirmed in future long term clinical trials regardless of whether it is related to on- or off-target effects.

Overall, for the patient and treating physician, the risk/benefit will be determined by both off-target and on-target effects and is based on safety and efficacy – regardless of target selectivity.

#### Inhibiting intracellular signaling may be as effective as targeting specific cell surface receptors

Inhibition of intracellular signaling molecules operating at critical nodes of pathways has always been an attractive approach to achieve a high magnitude and/or robust duration of therapeutic response in inflammatory immune mediated diseases [50]. Small molecules can directly target such intracellular pathways for which a large variety of candidate molecules exists; of those, kinases still remain the most attractive targets in rheumatology. Just as the biologic era came about as a consequence of major advances in protein engineering to allow the production of antibodies or engineered derivatives, advances in medicinal chemistry in the past two decades have also enabled the otherwise challenging development of very specific kinase inhibitors as therapeutic agents.

Kinases catalyze reversible phosphorylation, a fundamental mechanism for the regulation of protein function in various receptor-mediated processes, such as cell growth and differentiation in eukaryotic cells [51]. Many key immune receptors, including those responsible for driving inflammation, exert their effects through kinases and phosphorylation events. The human kinome comprises a total of 518 kinases and a large fraction of those functions downstream of cytokine, antigen and Fc-receptors, all representing potential targets for the treatment of rheumatic diseases.

More than 60 cytokines signal via the Janus kinase (Jak) and signal transducer and activator of transcription (STAT) pathways. Jak inhibitors have now been approved for the treatment of immune-mediated diseases, such as RA and myelofibrosis, and various Jak inhibitors are being developed and tested in a wide range of autoimmune diseases [52]. Although long term safety and efficacy data are not yet available for these inhibitors, the short term efficacy of tofacitinib, a Jak inhibitor in RA seems to be comparable to that of anti-TNF agents while side effects, such as infection, anemia and neutropenia, appear to be directly related to its mode of action [53].

Beyond Jaks, spleen tyrosine kinase (Syk) and Bruton tyrosine kinase (Btk) are also critical enzymes in the signaling pathways activated by immunological receptors involved in B and T cell function [50]. Kinases downstream of antigen and Fc-receptors also represent novel targets including members of the protein kinase C (PKC) family, mitogen-activated protein kinase (MAPK) family, the lipid kinase phosphoinositide 3-kinase (PI3K), protein kinase B (PKB, also known as Akt) and mammalian target of rapamycin (mTOR). Inhibitors of these proteins are currently being tested in preclinical models or clinical trials of various immune-mediated diseases [50]. Development of oral inhibitors of Jak and Syk are the most advanced and have already demonstrated clinical efficacy in RA.

Furthermore, not only kinases can be targeted by oral immune modulators. Examples include early studies in RA with a sphingosine-1-phosphate lyase inhibitor to modulate lymphocyte redistribution, or with the chemokine receptor-1 antagonist involved in osteoclast maturation, mobility and activation [54].

Given the large number of key effector proteins discovered in a wide variety of cell functions, together with the growing scientific understanding of the pathogenesis of immune mediated diseases, further small molecule based targeted immunomodulators will likely emerge in the near future.

#### Additional potential benefits of small molecules

Biologic agents and small molecules usually differ in their half-life, as well. Protein-like biologics currently used or tested in rheumatology usually have a longer half-life than small molecules. While such biologics require less frequent administration, they also need longer elimination; in cases where fast elimination is desirable, for example, during infections or before surgical interventions, the short half-life of small molecules may be of considerable clinical benefit.

Beyond the medical aspects, there are other factors that may have a positive effect on the overall cost-effectiveness of small molecules. Although it is too early to speculate about the long-term costs of small molecules, should these agents (requiring less complex and less expensive manufacturing) be significantly less expensive than biologics, this would certainly add significant value to the use of small molecules. Some small molecule drugs are also advantageous in terms of several logistical aspects, such as longer shelf life, no need for refrigeration and easier distribution in rural areas where infrastructure/experience with parenteral administration is limited.

## Summary

After a revolution in rheumatology by biologic therapy, novel intracellular targets have also emerged from an improved understanding of the immunopathogenesis of inflammatory rheumatic diseases. While biologics can modulate extracellular protein-protein interactions, intracellular processes can be more effectively targeted by small molecules. As a result, a number of small-molecule anti-rheumatic agents have been developed and many of those have progressed to preclinical stages or human clinical studies. As compared to biologics, small molecules also differ in the route of administration, target selectivity and specificity in terms of safety and efficacy as well as development path and overall cost. Despite such major advances in rheumatology, still no ideal treatment exists as current therapies are not causal and no single monotherapy, either small molecule or biologic, is capable of inducing full remission in the majority of patients in most rheumatic diseases. True synergies exist when both classic small molecule approaches and protein therapeutic strategies are simultaneously applied. Combination therapy likely continues to be a key therapeutic approach for most rheumatic diseases.

Currently, several novel approaches with biologics as well as small molecules are being tested to target various immunopathogenic players in immune-mediated rheumatic diseases. In addition to the existing effective biologic agents, highly effective oral drugs also seem to emerge. The next several years will witness a real-life comparison of the clinical efficacy and safety of biologics and oral therapies in rheumatology and provide the answers eagerly awaited by the rheumatology community.

## Competing interests

Peter Gergely is an employee of Novartis. The other two authors declare that they have no competing interests.

## Authors’ contributions

All three authors contributed to the conception and design of the study, helped to draft the manuscript, and read and approved the final manuscript.
